# The Regulation by Phenolic Compounds of Soil Organic Matter Dynamics under a Changing Environment

**DOI:** 10.1155/2015/825098

**Published:** 2015-10-01

**Authors:** Kyungjin Min, Chris Freeman, Hojeong Kang, Sung-Uk Choi

**Affiliations:** ^1^School of Civil and Environmental Engineering, Yonsei University, Seoul 120-749, Republic of Korea; ^2^Department of Ecology and Evolutionary Biology, University of Kansas, Kansas Biological Survey, Lawrence, KS 66047, USA; ^3^School of Biological Sciences, University of Wales, Bangor LL57 2UW, UK

## Abstract

Phenolics are the most abundant plant metabolites and are believed to decompose slowly in soils compared to other soil organic matter (SOM). Thus, they have often been considered as a slow carbon (C) pool in soil dynamics models. Here, however, we review changes in our concept about the turnover rate of phenolics and quantification of different types of phenolics in soils. Also, we synthesize current research on the degradation of phenolics and their regulatory effects on decomposition. Environmental changes, such as elevated CO_2_, warming, nitrogen (N) deposition, and drought, could influence the production and form of phenolics, leading to a change in SOM dynamics, and thus we also review the fate of phenolics under environmental disturbances. Finally, we propose the use of phenolics as a tool to control rates of SOM decomposition to stabilize organic carbon in ecosystems. Further studies to clarify the role of phenolics in SOM dynamics should include improving quantification methods, elucidating the relationship between phenolics and soil microorganisms, and determining the interactive effects of combinations of environmental changes on the phenolics production and degradation and subsequent impact on SOM processing.

## 1. Introduction

Phenolics consist of more than one aromatic ring, bearing one or more hydroxyl functional groups. They originate from plant materials and industrial products/wastes, which enter the soil either as leachates or as particulate matter [1]. Once integrated into the soil, phenolics can control below-ground processes, including SOM decomposition [2–4] and nutrient cycling [5, 6]. Recently, Freeman et al. [7] have suggested that modification of phenolics in peatland has a potential as a geoengineering tool to capture C in terrestrial ecosystems.

In spite of theses multitude of studies, however, controversies remain on how phenolics decompose in soils, how they modify the rate of SOM decomposition, and how current environmental changes will influence the fate of phenolics in soils. Given that phenolics represent one of the most abundant components in soils [8, 9] and that they affect the cycling of key nutrients to plants and soil microorganism [1, 9], it is indispensable to investigate the mechanisms by which phenolics influence decomposition biotically and abiotically and the degree to which these mechanisms will vary in response to environmental changes. This review presents current knowledge about phenolics and their role in decomposition under various environmental changes and proposes areas of future research. This review covers the following six areas: (1) various structures and forms of phenolics in soils; (2) how to extract and measure phenolics in soil samples; (3) biodegradation of phenolics; (4) effects of phenolics on SOM decomposition; (5) effects of environmental changes, such as elevated CO_2_, warming, N deposition, and drought, on phenolics and decomposition; and (6) suggestions for future phenolics studies.

## 2. Structure and Form in Soils

**Figure 1:**
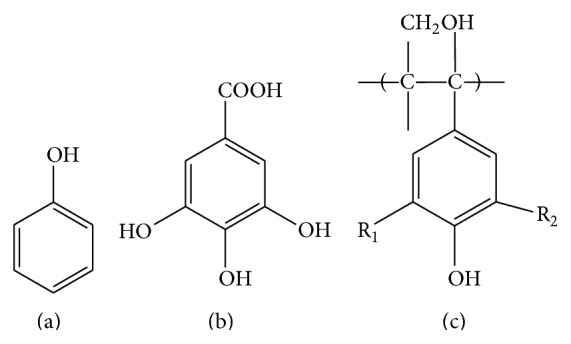
Chemical structures of several phenolics: phenol (a), the simplest structure of phenolic compound, phenolic acid (gallic acid) (b), and tannin (c).

Naturally, phenolic compounds are widely distributed throughout the plant kingdom, constituting up to 60% of plant dry mass [10]. Due to its loose definition (presence of at least one aromatic ring and hydroxyl group), more than 8,000 compounds have been classified as phenolics to date [11], encompassing simple, low molecular compounds to complex, highly polymerized compounds. Often, the number of aromatic rings and chemical structure are used to classify phenolics (Figure 1). For example, phenol, the simplest form of phenolics, has one aromatic ring with no extra carbon and belongs to class simple phenols. Class phenolic acids have a basic structure of C6-C1, including gallic acid, vanillic acid, and syringic acid. Lignin, one of the most common compounds in plants, is categorized as class lignins, exhibiting multiple combination of C6-C3 structure.

**Figure 2:**
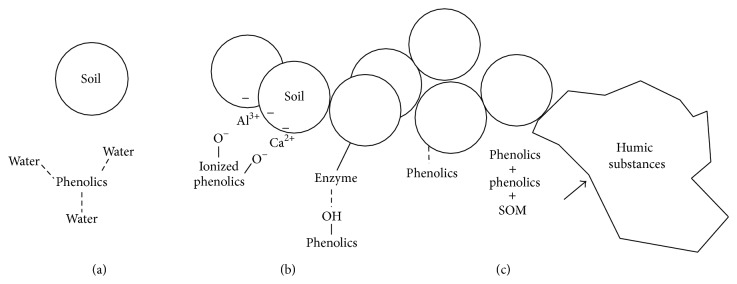
Various forms of phenolic compounds in soils. A dissolved form (a) where phenolics make multiple hydrogen bonds with water molecules surrounding them. A sorbed form (b) where phenolics are absorbed in soils and may detach from them reversibly through ionic, hydrogen, and hydrophobic bonds. A polymerized form (c) where phenolics consist of humic substances connected with other soil organic matter.

Phenolics in soils can exist as (1) a dissolved form, which moves freely in the soil solution, (2) a sorbed form, which reversibly binds to the soil particle or proteins, and (3) a polymerized form, consisting of humic substances (Figure 2). As many phenolics including phenolic acids and tannins are water soluble, they remain in solution between soil particles [15]. Reversible sorption of phenolics by soils occurs through hydrophobic, hydrogen, and ionic bond [8]. Humic substances, a stable polymer in soils, are generated by a polymerization of phenolics with other phenolics or soil organic matter [9].

Recent studies suggest that the form of phenolics, not their chemical structure, can influence their fate in soils [16–19], raising a question on conventional classification of phenolics into a slow, recalcitrant pool in C dynamics climate model [20]. For example, dissolved phenolics may have higher chance than sorbed or polymerized ones to encounter microorganisms in soil solution, allowing them to be processed quickly into simple, assimilable forms. In contrast, physically and chemically protected phenolics can persist longer than dissolved forms, providing feedbacks to SOM-decomposing microorganisms via changing soil pH, nutrient availability, and enzyme activities. Thus, caution is required to investigate the role of phenolics in SOM decomposition.

## 3. Extraction and Quantification of Phenolics

A variety of methods of extraction and quantification of phenolic compounds in soils have been established (Table 1). Solvents such as water, acetone, methanol, and citrate are widely used to extract phenolics. Blum [21] reported that soil samples extracted by water and citrate were suitable for estimating both free phenolic acids and sorbed phenolics. Arditsoglou and Voutsa [22] showed that acetone has higher extraction efficiency than methanol in aqueous samples. In contrast, Mukhopadhyay et al. [23] revealed that a mixture of methanol and water (6 : 4, v/v) was best to extract total phenolics and individual phenolic acids from black cohosh.

As the amount of phenolic compounds in soils can vary, the Folin-Ciocalteu assay is commonly used to determine the total amount of phenolic acids [24–26]. This assay is relatively simple compared to the CuO oxidation and the HPLC method. Thoss et al. [27] have compared 5 different methods to measure phenolic content in various freshwater samples. They concluded that a different pattern for each site originated from reactivity of phenolic materials and that Folin-Ciocalteu assay is the most appropriate for measurement of total phenolics. However, Ohno and First [28] pinpointed the limitations of the Folin-Ciocalteu assay that it is suited only for samples extracted by water, and interference by organic matter, such as sugars and aromatic amines, makes it impossible to precisely measure the amount of phenolic acids in citrate-extracted soils. In addition, the Folin-Ciocalteu assay was criticized for its low sensitivity [21].

Prior et al. [36] suggested correcting for nonphenolic compounds by using gallic acid as a reference for standardization. For quantification of highly polymerized lignin, gas chromatography (GC) followed by CuO oxidation is employed [37, 38]. CuO oxidation has the potential to be a powerful tool to estimate lignin content in soils as well as the degree of lignin decomposition [39]. Even though GC yields a high sensitivity, the low volatility of simple phenolic compounds requires a derivatization step, resulting in longer sample preparation [40]. Over recent decades, analysis of phenolics has been conducted via high performance liquid chromatography (HPLC) [41–44]. However, wide use of HPLC in ecological studies has been restricted by high cost and complicated process of operation.

## 4. Degradation of Phenolics

In soils, phenolics are mainly degraded by fungi (e.g., Basidiomycetes and Ascomycetes) and bacteria (e.g.,* Pseudomonas*). These microorganisms release extracellular enzymes into soils that break down phenolic compounds (Table 2). Phenolics-degrading enzymes are often named as phenol oxidase or peroxidase, according to their electron acceptor [45]. Both enzymes cause nonspecific oxidation of phenolic compounds, consuming oxygen and hydrogen peroxide as an electron acceptor, respectively.

Environmental factors, such as soil pH, temperature, oxygen, and substrate, can affect the degradation of phenolics. Contrary to the relatively low optimal pH of purified enzymes in laboratory conditions (Table 2), Sinsabaugh [45] found that there is a positive relationship between phenolics-degrading enzyme activities and soil pH across ecosystems. Likewise, Pind et al. [24] have reported that phenol oxidase activity increases as pH of peat soils increases. Regarding temperature, phenol oxidase showed no clear relationship [46–48] in the field conditions probably due to the interactive effecs of oxygen availability at different temperatures. However, purified phenol oxidase increased its decay of L-DOPA, a proxy of phenolics in lab conditions, at a temperatture of 5–25°C [49]. As phenol oxidase uses oxygen as an electron acceptor, its activity is proportional to oxygen concentration [24]. The relationship between the activity of phenol oxidase and phenolics concentration in natural ecosystem is not clear, as conflicting evidence is currently present. While some studies reported a positive relationship [2, 38, 50, 51], still others demonstrated contradictory results, reporting a negative or inverse relationship [25, 52–54] or no relationship [55, 56]. Such diverse responses, however, should be interpreted with care. In case of soil systems with a large amount of phenolics such as peat matrix, higher phenol oxidase in soil results in higher phenolic content in pore water as a product of enzyme action on peat, resulting in a positive relationship between phenol oxidase and phenolics. However, if correlation analysis was conducted between phenol oxidase in soils and phenolics in soil matrix or soil extract in mineral soils such forest soils, a negative correlation has often been reported because here phenolics may represent an enzyme substrate rather than a product. Another possibility is the dual functions of phenol oxidase. For example, Burke and Cairney [57] pointed out that mycorrhizal laccases can mediate both in depolymerization and polymerization and that, without the knowledge of redox mediators for these enzymes, predicting the direction of phenolics processing may be difficult.

Degradation of phenolics is usually reported by several groups to be slower than the degradation of other SOM fractions. The litter bag experiment demonstrated that labile compounds in litter such as carbohydrates and proteins were preferentially decomposed over phenolic compounds [3, 58]. As such, phenolic concentrations have been useful in predicting the rate of litter degradation [59, 60]. Yet, as stated above in Section 2, phenolics can decompose fast in certain conditions. Soluble phenolics and tannins degraded to 36~50% of the initial content in the litter bag experiment [61, 62]. Degradation of lignin, determined by the acid to aldehyde ratioin CuO oxidation products (see Table 1), was dominant over degradation of other SOM in forest soils [63]. ^14^C-labelling revealed that 56~68% of lignin from maize was transformed into CO_2_ during 6 months of a laboratory incubation [64].

## 5. Effects of Phenolics on Other SOM Decomposition

Effects of phenolics on SOM decomposition have been studied directly (i.e., litter bag) or indirectly (i.e., microbial biomass, extracellular enzyme activity, and heterotrophic respiration). Generally, phenolics reduced the rate of litter/SOM decomposition [65, 66]. Moreover, phenolic acids released by* Sphagnum* in peatlands suppressed bacterial and fungal growth [67, 68]. Even low concentrations of phenolics in peat homogenates have been noted to inhibit the activity of *β*-glucosidase, phosphatase, sulphatase, chitinase, and xylosidase by 21, 15, 32, 18, and 14%, respectively [69]. In addition, dissolved organic matter containing phenolic compounds from peat samples decreased CO_2_ production in anaerobic conditions [70]. Likewise, the rate of litter decomposition was shown to be inversely proportional to the phenolic content in litter [61].

**Figure 3:**
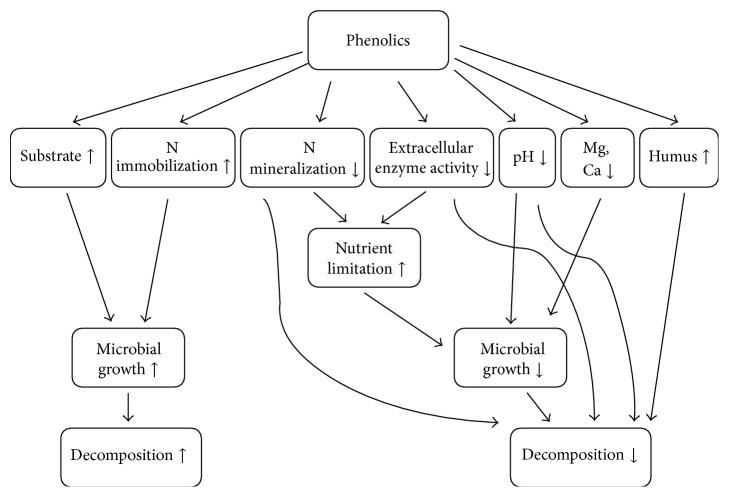
Effects of phenolics on the rate of soil organic matter decomposition.

As illustrated in Figure 3, the inhibition of decomposition by phenolics can occur via (1) formation of covalent bonds with proteins, decreasing N mineralization and enhancing N limitation to microorganisms [71], (2) oxidation of other phenolics, leading to humus formation [72], (3) suppression of microbial growth by lowering pH [73], (4) deprivation of metal ions by their high cation-exchange capacity [74], or (5) a formation of phenolic-enzyme complex, inactivating decomposition activity [75]. Yet, there are several studies beyond this simple negative relationship between phenolics and decomposition. Fierer et al. [76] found out that low molecular phenolic compounds and some tannins could serve as a labile substrate, promoting microbial biomass. Müller et al. [77] showed that lignin-derived phenolic compounds induced cellulase production, suggesting their potential to enhance decomposition. Significant reduction in SOM content was also observed after phenolics were added [78]. In agreement with this finding, phenolic concentrations have been reported to be positively correlated to CO_2_ release from soil [79] or litter [66].

Opposing reviews on the effect of phenolics on SOM decomposition argue further studies on the relationship between the forms and the roles of phenolics on decomposition and clearer terminology, as a wide range of molecules are defined as phenolics. In general, simple phenolics, such as phenolic acids, appear to increase decomposition, while complex phenolics decrease decomposition. Hoostal and Bouzat [80] showed that microbial extracellular enzyme activities were dependent on the source and composition of phenolics, rather than the absolute quantities of phenolics.

## 6. Effects of Environmental Changes on Phenolics

So far, several studies have aimed at elucidating how environmental changes such as elevated CO_2_, warming, N deposition, and drought may affect phenolic production from plant tissues, subsequent degradation in soils, and SOM decomposition.

Elevated CO_2_ usually increases phenolic concentrations in plants (Table 3). In field CO_2_ enrichment experiments, phenolic compounds in plant tissues, such as leaves, needles, stems, and rhizomes, increased by 11–182% [81–84]. Elevated CO_2_ can increase carbon supply and nutrient (e.g., N) stress in trees, resulting in decreased carbon demand. Such change is known to accelerate the accumulation of total nonstructural carbohydrates and the synthesis of carbon-based secondary or structural compounds [81]. Change in the concentration of phenolics from plant tissues may impart its effect on downstream processes including SOM decomposition. For example, Siegenthaler et al. [58] found that elevated CO_2_ induced a production of phenolic-rich litters, resulting in declining SOM decomposition. Effects of elevated CO_2_ on phenolic production in wetlands including peatlands have been extensively studied because wetlands are one of the key sources of DOC and phenolics to aquatic ecosystems. For example, elevated CO_2_ increased DOC and phenolic leaching from wetlands [69, 85], which may decrease hydrolase activities [2]. However, some studies have reported a faster degradation of phenolics at elevated CO_2_. After 559 days of litter bag incubation, lignin loss from Mongolian oak fine roots was 13% faster in the elevated CO_2_ chamber than in the ambient chamber, which was attributed to a 10% increase in phenol oxidase activity compared to the control nontreated group [86]. Moreover, phenolic compounds in an ombrotrophic bog decreased by 15.4% at elevated CO_2_ compared to control [87], suggesting that elevated CO_2_ may accelerate phenolic degradation. It appears that elevated CO_2_ often increases the total amount of carbon supplied to below-ground microorganisms and may induce “priming” effects to accelerate the decomposition of old or recalcitrant organic matter. Norby et al. [88] studied the effects of elevated CO_2_ on litter chemistry and decomposition rates in upland vegetation and demonstrated that elevated CO_2_ does increase lignin content in leaf litter significantly, but there is no significant effect on decomposition rate. In summary, further investigation is warranted on the effects of increasing phenolics on decomposition because of the involvement of other factors such as vegetation types, ecosystem types, nutrient availability, and changes in other factors (e.g., temperature and water availability).

Rising temperature is expected to be accompanied by an increase in atmospheric CO_2_ concentration. Few studies have measured the effect of warming on phenolic production and degradation, with an emphasis on whole organic matter decomposition. Unlike the rather unidirectional influences of elevated CO_2_, warming has various effects on the production of phenolics (Table 3). Increases in temperature have led to both an increase [89] and a decrease [90] in phenolic production. Warmer conditions usually accelerate biochemical reactions and may result in lowering production of secondary metabolites because plant growth would be enhanced. In fact, Zvereva and Kozlov [91] have reported lower phenolic contents under warming conditions than control in their meta-analysis. However, interactive or simultaneous effects of elevated CO_2_ and warming in relation to phenolics production have not been reported [83, 90] because two effects often negate each other [91].

N enrichment was studied in terms of atmospheric N deposition and fertilizer additions. Many studies suggest that phenolic concentrations are unchanged after N enrichment [92–94]. Extracellular enzymes, such as phenol oxidase and peroxidase, have been widely used for estimating the rates of phenolic degradation and SOM decomposition with N additions. Often, N enrichment decreases phenol oxidase [85, 95], while hydrolases are often activated. Sinsabaugh [45] reviewed that responses of phenol oxidase to N enrichment can differ by the types of ecosystem determined, as it decreases its activity in the forest and increases it in grassland or agricultural system. These contrasting results may originate from the initial lignocellulose contents in litter. In contrast, Bragazza et al. [96] have reported that N deposition can accelerate carbon release from peat bogs by activating phenol oxidase.

Global climate change models often predict increases in frequency and intensity of drought. Such changes can affect water availability in terrestrial ecosystems and water levels in wetlands. In wetlands, the effects of drought on nutrient cycling have drew much attention due to their close association with water. Most studies reported that drought increases the activity of phenol oxidase, implying stimulated decomposition [87, 97–99]. On the other hand, reduction in phenol oxidase activity was also found in peatland and heathland in response to simulated drought [4, 100]. Toberman et al. [100] suggested that initial water content in soils may be responsible for these contrasting responses and that a hyperbolic relation exists between water content and phenol oxidase.

## 7. Phenolics for Carbon Storage

Changes in the concentration, form, and decay rate of phenolics in response to climate can guide to better sequester terrestrial C. Recently, Freeman et al. [7] have proposed that enhanced carbon storage in ecosystems, particularly in peatlands, is feasible by modifying phenolic contents which inhibit decomposition of organic matter by a mechanism called “enzymic latch” [113, 114]. They proposed that increases in phenolic content in peat ecosystems can be achieved either by increased expression of phenolic inhibitors from peatland plants or by enhancement of enzyme latch by physicochemical modification. Furthermore, it is widely known that phenolic content can be enhanced by modifying pyrolysis conditions such as temperature, pyrolysis time, substrate, and oxygen supply for biochar preparation [115]. As such, we propose that addition of biochar with high phenolics content represents a further approach to stabilize SOM in terrestrial ecosystems by inhibiting enzyme activities [116].

## 8. Future Studies Suggested

Studies of the ecological significance of phenolics have been conducted extensively since 1980, contributing significant understanding of their production, quantification, degradation, and effect on decomposition. As a secondary metabolite, phenolics have a range of structures and forms, with different reactivity. As such, application of appropriate methods for extraction and measurement must be applied according to the aims of each study. Assays to assess the activities of phenol oxidase and peroxidase have been developed to predict the degree and the direction of phenolic degradation.

However, there still remains controversy over how phenolics influence soil C cycling and how they are likely to respond to anticipated global environmental changes. For example, a larger supply of phenolics by global climate change may result in either faster or slower decomposition depending on the wider environmental conditions (Figure 3). Further, we conclude that the opposing trends of the effect of phenolics on SOM decomposition may be attributed to an insufficiently refined definition of the term “phenolics” or to the lack of information on redox mediators that control extracellular enzyme activities. We, therefore, propose that further studies are needed to understand fate of phenolics in response to simultaneous environmental changes with far higher resolution than in current practice. ^13^C labeling may be an appropriate tool to elucidate phenolic turnover in soils. Additionally, molecular approaches aiming at specific genes for phenol degrading enzymes must be considered. Enhancing our knowledge about the role of phenolics following environmental change will facilitate a better understanding of nutrient dynamics in soils. Ultimately, such information can also be applied to techniques for carbon sequestration in terrestrial ecosystems by slowing down decomposition processes.

## Figures and Tables

**Table 1:** Methods to quantify phenolic compounds.

Assay	Types of phenolics	Description	Reference
Folin-Ciocalteu assay	Total phenolic acids	An assay based on electron transfer (ET) in which oxidation of phenolics by Folin-Cioalteu reagent gives a colored product at 750 nm	[12]

CuO oxidation-GC	Lignin-derived phenolics	A method in which oxidation of lignin by cupric oxide yields single-ring phenol compounds (vanillyl-, syringyl-, and p-coumaryl units), followed by gas chromatography Also, the acid to aldehyde ratio can be used to estimate the state of decomposition of lignin	[13]

HPLC	Individual	A separation technique in which a mixture of phenolics produces different retention times depending on their affinity to the stationary phase	[14]

**Table 2:** Extracellular enzymes involved in phenolics degradation in soils.

Enzyme	Microorganism	Optimum condition		Reference
		pH	Temperature	
Lignin peroxidase	*Phanerochaete chrysosporium *	2.5		[29]
	*Phanerochaete chrysosporium *	4.2	34	[30]

Manganese peroxidase	*Phanerochaete chrysosporium *	4.5	32	[31] [30]
	*Phanerochaete sordida *	4.5~5.0		[32]

Laccase	*Trametes versicolor *	2.0		[29]
	Basidiomycete PM1	4.5	80	[33]
	*Pycnoporus sanguineus *	3~5	55	[34]

Phenol oxidase	*Termitomyces albuminosus *	2.3		[35]

**Table 3:** Effects of environmental changes on phenolics and decomposition.

Environmental changes	Phenolics production	Phenolics degradation	Decomposition	References
CO_2_	+/−			[83]
	+			[84]
	×			[101]
	+			[102]
	+			[103]
	+/×			[104]
	×	−		[105]
	+		−	[58]
	+			[82]
	+			[90]
	+			[81]
		+		[86]
		+	+	[87]

Warming	−			[90]
	×			[83]
		×		[106]
	+/−			[107]
	+			[89]
		−		[108]

N deposition	×	+/−		[105]
	+		−	[58]
			+/−	[45]
	×/−			[94]
	×/−			[92]
	−			[93]

Drought		+	+	[87]
	−			[109]
			−	[100]
		×/−	−	[100]
	+			[110]
			+	[98]
		+	+	[99]

CO_2 _× Warming	×			[90]
	×/−			[83]
		−		[108]

CO_2 _× N deposition		−		[111]
		−		[105]
		−		[58]

CO_2 _× Drought		×		[87]

Warming × N deposition	−			[112]

+: stimulation, −: inhibition, and ×: no effect or interaction.
